# Role of protease‐activated receptor‐1 (PAR‐1) in the glomerular filtration barrier integrity

**DOI:** 10.14814/phy2.15343

**Published:** 2022-08-03

**Authors:** Ajay Medipally, Min Xiao, Laura Biederman, Anjali A. Satoskar, Iouri Ivanov, Brad Rovin, Samir Parikh, Bryce A. Kerlin, Sergey V. Brodsky

**Affiliations:** ^1^ Departments of Pathology The Ohio State University Wexner Medical Center Columbus Ohio USA; ^2^ Departments of Medicine The Ohio State University Wexner Medical Center Columbus Ohio USA; ^3^ Department of Pediatrics Nationwide Children’s Hospital Columbus Ohio USA; ^4^ Center for Clinical and Translational Research Abigail Wexner Research Institute Nationwide Children’s Hospital Columbus Ohio USA

**Keywords:** anticoagulant related nephropathy, glomerular filtration barrier, PAR‐1, renal pathology

## Abstract

Protease‐activated receptors (PAR) play an important role in the regulation of cellular function by the coagulation system, and they are activated by thrombin. PAR‐1 is expressed in both endothelial cells and podocytes in the kidney. The role of PAR1 in the maintenance of the glomerular filtration barrier is not clear. Anticoagulant‐related nephropathy (ARN) is a kidney disease with glomerular hematuria and red blood cell tubular casts. We validated 5/6 nephrectomy (5/6NE) in rats as a model of ARN and had demonstrated that direct thrombin inhibitor (dabigatran) induces ARN. The aim of this study was to investigate the role of PAR‐1 in the ARN pathogenesis. 5/6NE rats were treated with dabigatran (150 mg/kg/day), PAR‐1 inhibitor SCH79797 (1 and 3 mg/kg/day) and PAR‐1 agonist TFLLR‐NH2 (0.25 and 0.50 µmol/kg/day) for 7 days. Serum creatinine and hematuria were assessed daily. Kidney morphology was evaluated at the end of the study. In 5/6NE rats treated with either dabigatran or combination with a PAR‐1 modulator, there was an elevation in serum creatinine, glomerular hematuria, red blood casts in the tubules, and acute tubular epithelial cell injury. Interestingly, both PAR‐1 modulators in a dose‐depended manner had similar effects on the serum creatinine levels and hematuria as those of dabigatran. Dabigatran‐induced increase in the systolic blood pressure was not affected by PAR‐1 modulators. In conclusion, the normal function of PAR‐1 is crucial to maintain the glomerular filtration barrier integrity. Either activation or blockage of PAR‐1 leads to glomerular hematuria and subsequent acute tubular epithelial cell injury.

## Graphical abstract

Red blood cell tubular casts in animals treated with PAR‐1 modulators with and without direct thrombin inhibitor dabigatran.

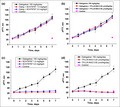

## 1 INTRODUCTION

Protease‐activated receptors (PAR) are membrane G‐protein‐coupled receptors that are expressed in many different cell types. Four PAR receptors are recognized. Among those, PAR1 and PAR2 are expressed in glomerular endothelial cells, mesangial cells, and kidney tubular epithelial cells in both humans and mice; all four types of PAR are expressed in human podocytes, as well as in rat and mouse podocytes (Harris et al., 2013; Oe et al., 2021; Sharma et al., 2017a). PARs undergo cleavage by proteases at the N‐terminus and the revealed neo‐N‐terminus activates the receptor as a tethered ligand. It has been demonstrated that PARs are activated by specific coagulation proteases. Specifically, Tissue Factor (TF) and Factor VIIa (FVIIa) complex activates PAR2, factor Xa activates both PAR1 and PAR2, and thrombin activates PAR1, PAR3, and PAR4 (Camerer et al., 2000; Coughlin, 2001, 2005; Rothmeier & Ruf, 2012). PARs play a significant role in the regulation of angiogenesis and fibrosis (Brodsky, 2002). However, their role in the regulation of glomerular filtration barrier (GFB) function remains unclear.

Anticoagulant‐related nephropathy (ARN) is characterized by glomerular hemorrhage and acute kidney injury with tubular red blood cell (RBC) casts, and it can occur with all anticoagulant classes (Brodsky et al., 2011, 2017, 2018, 2019). 5/6 nephrectomy rats treated with vitamin K inhibitors or direct thrombin inhibitor (dabigatran) develop glomerular hemorrhage with occlusive RBC casts and serve as an animal model of ARN (Ozcan et al., 2012; Ryan et al., 2014; Ware et al., 2011). We had demonstrated that inhibition of PAR‐1 by SCH79797 results in glomerular hemorrhage and acute kidney injury in 5/6 nephrectomy rats (5/6NE), effects similar to those of direct thrombin inhibitor dabigatran and mimicking human anticoagulant related nephropathy (Ryan et al., 2014). The aim of the current study is to investigate the synergic effects of dabigatran with PAR‐1 inhibition and activation in 5/6NE.

## 2 MATERIAL AND METHODS

The studies were approved by the Institutional Animal Care and Use Committees (IACUC) at the Ohio State University.

The 5/6 nephrectomy was performed in male Sprague Dawley rats (120–140 gm, the Charles River Laboratories, Wilmington, MA) under isoflurane/oxygen (1:5) anesthesia, as we described in prior studies (Ozcan et al., 2012; Ryan et al., 2014). A nephrectomy of the right kidney and resection of two‐thirds of the left kidney were performed simultaneously. Hemostasis was achieved by hemostatic sponges (Quick clot; Z‐medica Corporation, Wallingford, CT), which were removed before closure of the incision. The middle laparotomy wound was closed with a 4.0 proline, and the animals were kept at 12h/12h light/dark cycle on the standard rodent diet with free access to water. Osmotic minipumps (Alzet, Cupertino, CA, USA) were implanted under isoflurane/oxygen (1:5) anesthesia in the posterior neck.

All treatments began 3–4 weeks after the ablative surgery. The following study groups were used in the studies: (1) 5/6NE rats treated with 150 mg/kg/day dabigatran alone, (2) 5/6NE rats treated with dabigatran and PAR‐1 inhibitor SCH79797 (2 doses), (3) 5/6NE rats treated with dabigatran and PAR‐1 agonist TFLLR‐NH2 (Kawabata et al., 1999; Kawao et al., 2003) (2 doses), (4) 5/6NE rats treated with PAR‐1 inhibitor SCH79797 (2 doses) alone, (5) 5/6NE rats treated with PAR‐1 agonist TFLLR‐NH2 (2 doses) alone, (6) 5/6NE rats treated with vehicle were used as control.

Dabigatran etexilate (Boehringer Ingelheim Pharmaceuticals, Inc. Ridgefield, CT) was given daily by a gavage. PAR‐1 inhibitor SCH79797 was injected intraperitoneally in 20% DMSO twice a day (one‐half of the daily dose in each injection) as we described previously (Ryan et al., 2014). PAR‐1 agonist TFLLR‐NH2 was administered via osmotic minipump.

Hematuria was measured by Siemens Multistix 5 (Siemens Healthcare Diagnostics Inc, Tarrytown, NY) and recorded in a semiquantitative scale from 0 to 3, where score 0 is absent, 1+ is trace, 2+ is moderate, and 3+ is large. Serum creatinine was measured in tail blood; the samples (100 mcl) were collected via a tail puncture, as described previously (Ozcan et al., 2012; Ware et al., 2013). At the end of the study, the animals were sacrificed, and the remnant kidney was cut longitudinally into two halves for histology and other studies. Histology of the kidney was blindly evaluated by renal pathologists (SB, LB, AS) on 2–3 mcm sections of paraffin‐embedded tissue stained with hematoxylin and eosin (H&E).

Serum creatinine was measured based on the Jaffe reaction using a creatinine reagent assay kit (Raichem, San Marcos, CA) according to the manufacturer protocol, as reported previously (Ozcan et al., 2012; Ryan et al., 2014). In brief, 10 mcl of serum was mixed with 200 mcl of working reagent at 37°C in a 96‐well plate, and the absorbance was read at 510 nm at 60 and 120 s on a microplate reader (Molecular Devices, Sunnyvale, CA).

Activated partial thromboplastin time (aPTT) was measured by using a Fisher Scientific Thromboscreen 200 Hemostasis Analyzer (Fisher Scientific, Middletown, VA) based on the manufacturer protocol, as we described previously (Ryan et al., 2014). Briefly, tail blood was collected into a tube containing 3.8% sodium citrate in a ratio of 9:1. The blood was centrifuged at 3500 RPM for 10 min, 20 μl of plasma was placed in the incubation station with 20 μl of the aPTT reagent (Fisher scientific, Middletown, VA), and 20 μl of 0.025 M pre‐heated calcium chloride was added 3 min later. Clotting time was recorded in seconds.

Blood pressure was measured by a tail‐cuff using a blood pressure monitor (IITC Life Sciences Inc. Woodland Hills, CA), as we previously described (Ware et al., 2015). The systolic, diastolic, and mean blood pressures were determined using the Blood Pressure Data Acquisition Software (IITC Life Sciences Inc. Version 1.35).

### 2.1 Statistical analysis

Results are presented as mean ± standard deviation (SD) if not otherwise specified. Differences between two groups were analyzed by the two‐paired *t*‐test or two‐way ANOVA test, where applicable.

## 3 RESULTS

### 3.1 PAR‐1 modulators effects on serum creatinine levels

**FIGURE 1:**
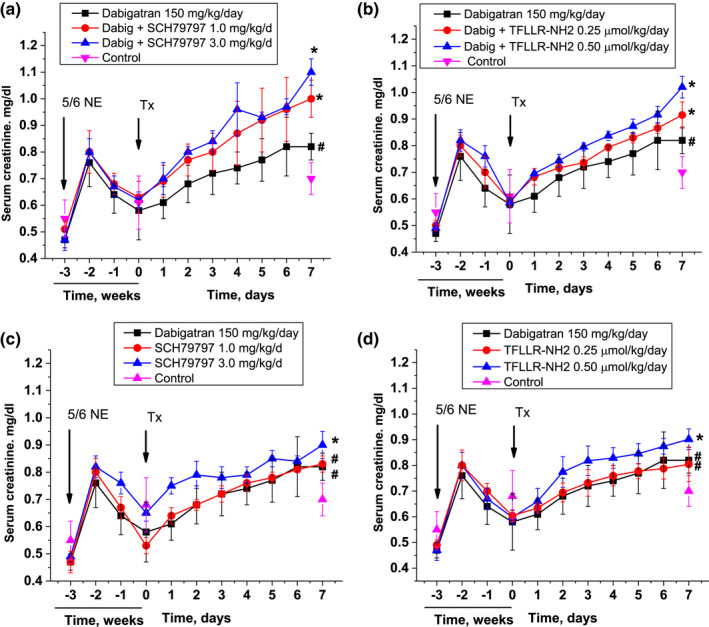
Changes in serum creatinine levels in 5/6 nephrectomy rats treated with direct thrombin inhibitor dabigatran and PAR‐1 modulators. (a) Changes in serum creatinine levels in 5/6NE rats treated with dabigatran alone (150 mg/kg/day) and in combination with PAR‐1 inhibitor SCH79797 (1.0 and 3.0 mg/kg/day). 5/6NE rats that receive vehicle are show as control. (b) Changes in serum creatinine levels in 5/6NE rats treated with dabigatran alone (150 mg/kg/day) and in combination with PAR‐1 agonist TFLLR‐NH2 (0.25 µmol/kg/day and 0.50 µmol/kg/day). 5/6NE rats that receive vehicle are show as control. (c) Changes in serum creatinine levels in 5/6NE rats treated with dabigatran (150 mg/kg/day) or only with PAR‐1 inhibitor SCH79797 (1.0 mg/kg/day and 3.0 mg/kg/day). 5/6NE rats that receive vehicle are show as control. (d) Changes in serum creatinine levels in 5/6NE rats treated with dabigatran alone (150 mg/kg/day) or only with PAR‐1 agonist TFLLR‐NH2 (0.25 µmol/kg/day and 0.50 µmol/kg/day). 5/6NE rats that receive vehicle are show as control. Treatment (Tx) started at 3 weeks after the ablative surgery (5/6NE). *p < 0.05 as compared to dabigatran alone. #*p* < 0.05 as compared to control.

Treatment with dabigatran (150 mg/kg/day) alone or in combination with PAR‐1 inhibitor SCH79797 (1 mg/kg/day and 3 mg/kg/day) or agonist TFLLR‐NH2 (0.25 µmol/kg/day and 0.50 µmol/kg/day) began at 3 weeks after the 5/6 nephrectomy, similar to previous studies (Ryan et al., 2014). Dabigatran alone and in combination with either PAR‐1 inhibitor or agonist resulted in a gradual increase in the serum creatinine levels by day 7 of the studies (Figure 1a,b). Both PAR‐1 inhibitor SCH79797 (Figure 1a) and PAR‐1 agonist TFLLR‐NH2 (Figure 1b) potentiated dabigatran effect on the serum creatinine in a dose‐depended manner (two‐way ANOVA test, *p* = 0.018 for SCH79797 and *p* = 0.0389 for TFLLR‐NH2). Serum creatinine in 5/6NE treated with 150 mg/kg/day dabigatran alone was 0.82 ± 0.05 mg/dl at day 7. Treatment with both dabigatran 150 mg/kg/day and PAR‐1 inhibitor SCH79797 1 mg/kg/day resulted in an increase in serum creatinine by day 7 to 1.00 ± 0.07 mg/dl (*p* = 0.004), and, when the SCH79797 dosage was 3 mg/kg/day with the same dose of dabigatran, the serum creatinine increased to 1.1 ± 0.05 mg/dl (*p* = 0.002) at day 7 of the combined treatment (Figure 1a).

Similarly, combined treatment with TFLLR‐NH2 0.25 µmol/kg/day and dabigatran 150 mg/kg/day resulted in serum creatinine of 0.92 ± 0.05 mg/dl (*p* = 0.0063) by day 7 of treatment, and combined treatment with TFLLR‐NH2 0.50 µmol/kg/day and dabigatran 150 mg/kg/day lead to an increase in serum creatinine to 1.02 ± 0.05 mg/dl (*p* < 0.001) by day 7 (Figure 1b).

Interestingly, when PAR‐1 inhibitor SCH79797 and PAR‐1 agonist TFLLR‐NH2 were used alone, their effects on the serum creatinine levels were similar to those of dabigatran alone (Figure 1c,d). Thus, we confirmed our previous observations that SCH79797 in a dose‐dependent manner increases serum creatinine in 5/6NE rats (Ryan et al., 2014), and also show that the same is true for TFLLR‐NH2. Treatment with 1 mg/kg/day of SCH79797 resulted in a similar increase in the serum creatinine as dabigatran 150 mg/kg/day alone. However, when SCH79797 was used in the dose 3 mg/kg/day, serum creatinine increase at day 7 was higher than in dabigatran 150 mg/kg/day (Figure 1c). Serum creatinine in rats treated with dabigatran 150 mg/kg/day was 0.82 ± 0.02 mg/dl versus 0.90 mg/dl in 5/6NE rats treated with 3 mg/kg/day SCH79797, *p* = 0.0211. Similarly, TFLLR‐NH2 alone in a dose‐dependent manner also resulted in serum creatinine increase (Figure 1d). When 5/6NE rats were treated with 0.50 µmol/kg/day of TFLLR‐NH2, the increase in serum creatinine was higher than in 5/6NE rats treated with 150 mg/kg/day of dabigatran alone (1.01 ± 0.02 mg/dl vs. 0.81 ± 0.02 ng/dl, *p* < 0.0001).

### 3.2 PAR‐1 modulators effects on hematuria

**FIGURE 2:**
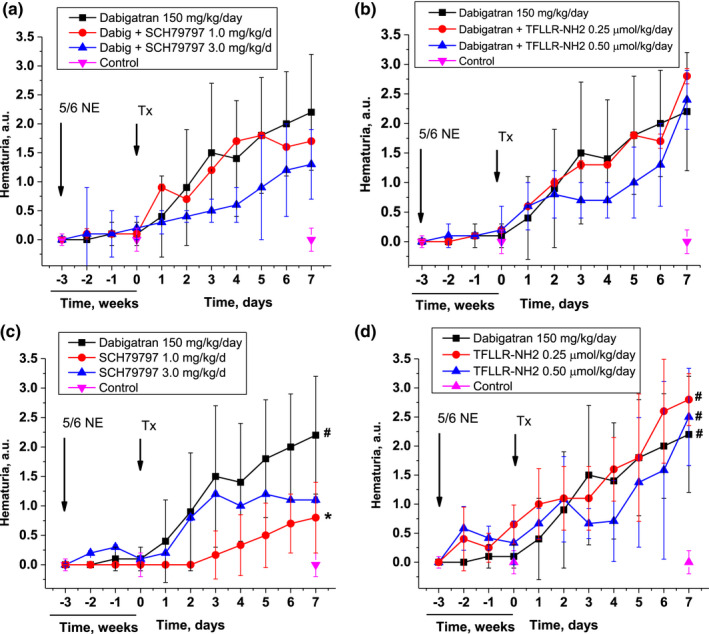
Changes in hematuria in 5/6 nephrectomy rats treated with direct thrombin inhibitor dabigatran and PAR‐1 modulators. (a) Changes in hematuria in 5/6NE rats treated with dabigatran alone (150 mg/kg/day) and in combination with PAR‐1 inhibitor SCH79797 (1.0 mg/kg/day and 3.0 mg/kg/day). 5/6NE rats that receive vehicle are show as control. (b) Changes in hematuria in 5/6NE rats treated with dabigatran alone (150 mg/kg/day) and in combination with PAR‐1 agonist TFLLR‐NH2 (0.25 µmol/kg/day and 0.50 µmol/kg/day). 5/6NE rats that receive vehicle are show as control. (c) Changes in hematuria in 5/6NE rats treated with dabigatran (150 mg/kg/day) or only with PAR‐1 inhibitor SCH79797 (1.0 mg/kg/day and 3.0 mg/kg/day). 5/6NE rats that receive vehicle are show as control. (d) Changes in hematuria in 5/6NE rats treated with dabigatran alone (150 mg/kg/day) or only with PAR‐1 agonist TFLLR‐NH2 (0.25 and 0.50 µmol/kg/day). 5/6NE rats that receive vehicle are show as control. Treatment (Tx) started at 3 weeks after the ablative surgery (5/6NE). *p < 0.05 as compared to dabigatran alone. #*p* < 0.05 as compared to control. Hematuria is scored in a semiquantitative scale from 0 to 3, where score 0 is absent, 1+ is trace, 2+ is moderate and 3+ is large.

Treatment with dabigatran (150 mg/kg/day) alone and in combination with either PAR‐1 agonist TFLLR‐NH2 (dosed at 0.25 µmol/kg/day and 0.50 µmol/kg/day) or inhibitor SCH79797 (dosed at 1 mg/kg/day and 3 mg/kg/day) resulted in gradual development of hematuria in 5/6NE rats (Figure 2a,b). Addition of either PAR‐1 inhibitor or agonists did not significantly modify the hematuric effects of dabigatran (two‐way ANOVA of changes in hematuria with the addition of SCH79797, *p* = 0.3141; two‐way ANOVA of changes in hematuria with addition of TFLLR‐NH2, *p* = 0.4578), Figure 2a,b. Similar to our previous observations, PAR‐1 antagonist SCH79797 alone in a dose‐dependent manner increased hematuria in 5/6NE rats, though to a lesser degree than dabigatran alone (Figure 2c).

**FIGURE 3:**
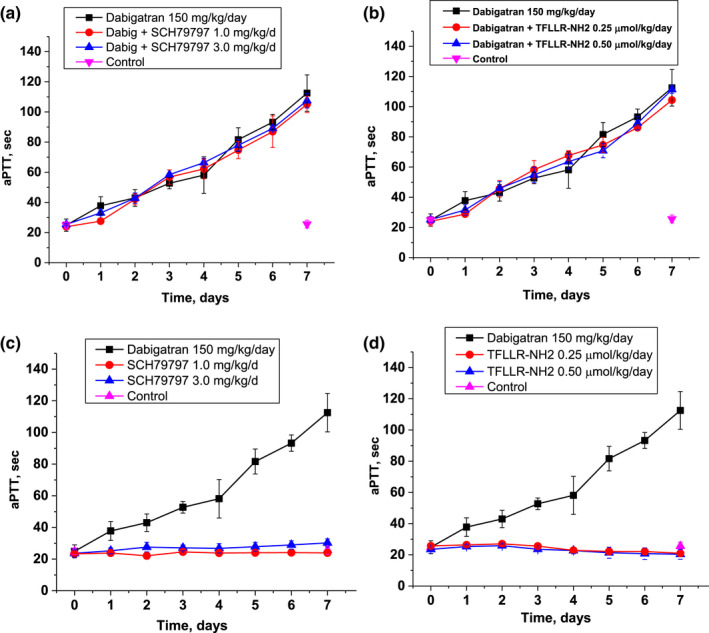
Histologic findings in 5/6 nephrectomy rats treated with direct thrombin inhibitor dabigatran and PAR‐1 modulators. (a) A representative image of red blood cell (RBC) tubular casts and acute tubular epithelial cell injury in a 5/6NE rat treated with 150/mg/kg/day dabigatran. Hematoxylin and eosin, magnification 100x. (b) percentage of RBC tubular casts in 5/6NE rats treated with direct thrombin inhibitor dabigatran and PAR‐1 modulators. (c) a representative image of negative iron stain in the tubules in a 5/6NE rat treated with 150 mg/kg/day dabigatran. Prussian Blue iron stain, magnification 100×.

Histologically, 5/6NE rats treated with dabigatran and PAR‐1 modulators had RBC tubular casts (Figure 3a). Different treatments resulted in different numbers of RBC casts in the kidney, but the percentage of tubules containing RBC casts was similar to the semi‐quantitative hematuria data measured. Dabigatran alone and in combination with TFLLR‐NH2 had the highest percentage of RBC tubular casts in the kidney. Animals that were treated with TFLLR‐NH2 alone also had RBC tubular casts, and the percentage was increased in a dose‐dependent manner. 5/6NE rats treated with dabigatran and SCH79797 had a lower percentage RBC tubular casts compared to 150 mg/kg/day dabigatran alone, though this decrease was not statistically significant. Treatment with SCH79797 alone also resulted in an increased number of RBC tubular casts in 5/6NE rats (Figure 3b).

Iron stain was negative in the tubular epithelial cells in all animals regardless of the treatment (Figure 3c).

### 3.3 PAR‐1 modulators effects on coagulation and blood pressure

**FIGURE 4:**
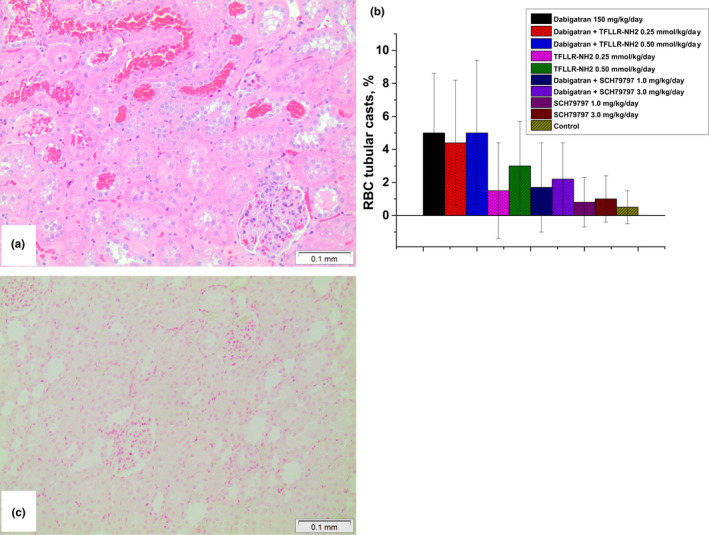
Changes in activated partial thromboplastin time (aPTT) in 5/6 nephrectomy rats treated with direct thrombin inhibitor dabigatran and PAR‐1 modulators. (a) Changes in aPTT in 5/6NE rats treated with dabigatran alone (150 mg/kg/day) and in combination with PAR‐1 inhibitor SCH79797 (1.0 and 3.0 mg/kg/day). 5/6NE rats that receive vehicle are show as control. (b) Changes in aPTT in 5/6NE rats treated with dabigatran alone (150 mg/kg/day) and in combination with PAR‐1 agonist TFLLR‐NH2 (0.25 and 0.50 µmol/kg/day). 5/6NE rats that receive vehicle are show as control. (c) Changes in aPTT in 5/6NE rats treated with dabigatran (150 mg/kg/day) or only with PAR‐1 inhibitor SCH79797 (1.0 and 3.0 mg/kg/day). 5/6NE rats that receive vehicle are show as control. (d) Changes in aPTT in 5/6NE rats treated with dabigatran alone (150 mg/kg/day) or only with PAR‐1 agonist TFLLR‐NH2 (0.25 µmol/kg/day and 0.50 µmol/kg/day). 5/6NE rats that receive vehicle are show as control. Treatment (Tx) started at 3 weeks after the ablative surgery (5/6NE). *p < 0.05 as compared to dabigatran alone. #*p* < 0.05 as compared to control.

Dabigatran 150 mg/kg/day resulted in a steady increase in aPTT in 5/6NE rats, similar to our earlier observations, Figure 4a‐d (Ryan et al., 2014). Neither PAR‐1 modulator affected the anticoagulation effects of dabigatran (Figure 4a,b), and neither resulted in aPTT changes when administered alone (Figure 4c,d) as compared to control.

**FIGURE 5:**
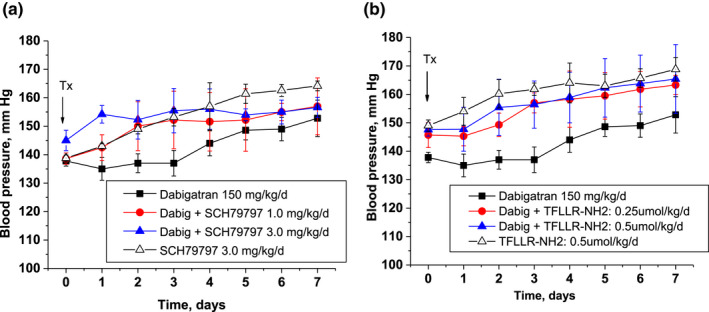
Changes in systolic blood pressure in 5/6 nephrectomy rats treated with PAR‐1 modulators. (a) Changes in systolic blood pressure in 5/6NE rats treated with dabigatran alone (150 mg/kg/day) and in combination with PAR‐1 agonist TFLLR‐NH2 (0.25 µmol/kg/day and 0.50 µmol/kg/day) or with TFLLR‐NH2 0.50 µmol/kg/day alone. (b) Changes in systolic blood pressure in 5/6NE rats treated with dabigatran (150 mg/kg/day) or only with PAR‐1 inhibitor SCH79797 (1.0 and 3.0 mg/kg/day) or with SCH79797 3.0 mg/kg/day alone.

Systolic blood pressure was increased after treatment with 150 mg/kg/day dabigatran, similar to our prior observations (Ware et al., 2015). Treatment with both PAR‐1 modulators did not significantly affected dabigatran‐induced blood pressure increase when they were administered with dabigatran, nor they changes systolic blood pressure when were used alone, even though there was a trend in higher systolic blood pressure when a PAR‐1 modulator was used in combination with dabigatran (Figure 5a,b). Diastolic blood pressure was not affected by any combination of treatments (data not shown). Body weight changes in 5/6NE rats were not affected by either of the treatment as compared to control.

## 4 DISCUSSION

In the current study, we report effects of PAR‐1 modulators on the hematuria and serum creatinine levels in 5/6NE rats. We had demonstrated previously that in 5/6NE rats that are treated with different anticoagulants including vitamin K antagonists (Ozcan et al., 2012; Ware et al., 2011) and direct thrombin inhibitor (Ryan et al., 2014) results in an animal model that mimics human ARN (Brodsky et al., 2010, 2017, 2018, 2019; Kadiyala et al., 2012). Herein we demonstrate that modulation of PAR‐1 by either PAR‐1 inhibitor (SCH79797) or agonist (TFLLR‐NH2) also results in increased serum creatinine, glomerular hematuria, RBC casts in the tubules, and acute tubular epithelial cell injury without changes in coagulation. This data suggest an important role of PAR‐1 in the maintenance of the GFB integrity.

The GFB is formed by the endothelium, glomerular basement membrane, and podocytes. Endothelial cell injury in rats treated with warfarin has been shown by electron microscopy (Kahn et al., 1971), suggesting the endothelium may play an important role in the response to anticoagulants and therefore anticoagulant nephropathy. Several types of thrombin receptors are expressed on endothelial cells, including PAR‐1. PAR‐1 is a G protein‐coupled receptor that participates in the regulation/protection of the endothelium by blocking cytokine signaling, adhesion molecule expression, vascular permeability, apoptosis, and leukocyte migration and adhesion (Coughlin, 2001, 2005). In vitro studies indicate that PAR‐1 activation stimulates the RhoA/Rho kinase signaling pathway, leading to phosphorylation of myosin light chain, formation of actin stress fibers, and changes in endothelial monolayer integrity (Carlile‐Klusacek & Rizzo, 2007). It also has been demonstrated that PAR‐1 regulates endothelial permeability by disassembling claudin‐5 from tight junctions (Kondo et al., 2009). PAR‐1 activation on endothelial cells can have opposing biologic effects, resulting in either endothelial cell barrier disruption when activated by high concentration of thrombin or barrier‐protective effect when a low concentration of thrombin and activated protein C (APC) is involved (Feistritzer & Riewald, 2005). It has been shown that APC—PAR‐1 signaling prevents vascular leakage in a murine sepsis model (Niessen et al., 2009). Coagulation factor Xa also regulates endothelial cell barrier integrity via PAR‐1 transactivation of PAR‐2 receptors (Feistritzer et al., 2005; Lin et al., 2013; Soh et al., 2010), suggesting that factor Xa inhibitors may also affect glomerular endothelial barrier integrity. Little is known regarding PAR‐1 expression changes in CKD patients (Izmirly et al., 2009). However, the results we presented suggest that, at least in ablative nephropathy, PAR‐1 modulation may potentiate the severity of the disease. Further research is necessary to elucidate the role of PAR‐1 in the chronic phase of the disease as well as in other causes of chronic kidney disease.

Although both PAR‐3 and PAR‐4 are also endothelial thrombin receptors, they are only expressed at low levels in kidney endothelial cells and most likely do not play a significant role in thrombin‐mediated ARN pathogenesis (Madhusudhan et al., 2012; Scridon et al., 2019).

Both SCH79797 and TFLLR‐NH resulted in elevation in the systolic blood pressure and, presumably, change the glomerular filtration rate (GFR). The GFR changes probably did not play a significant role in the effects of PAR‐1 modulators, because we had demonstrated that changes in GFR did not change effects of dabigatran on hematuria and serum creatinine in 5/6NE rats (Medipally et al., 2021). Dabigatran itself did not change GFR in 5/6NE rats.

Our previous study with PAR‐1 antagonist SCH79797 (Ryan et al., 2014) and current observations strongly suggest that PAR‐1 plays an important role in the pathogenesis of glomerular hematuria. Recent discoveries indicate the important role of thrombin and its receptors in the regulation of podocyte function and proteinuria (Sharma et al., 2017b).

In conclusion, we unveiled that the normal function of PAR‐1 is crucial to maintain the GBF function. Either activation or blockage of PAR‐1 results in glomerular hematuria and acute tubular epithelial cell injury. This data provide important insights on the pathogenesis of GBF dysfunction.

## CONFLICTS OF INTEREST

The Authors declare no conflict of interest.

## ETHICS STATEMENT

The studies were approved by the Institutional Animal Care and Use Committees (IACUC) at the Ohio State University.
